# Sex Differences in Sleep and Physical Activity Patterns in Autism Spectrum Disorder

**DOI:** 10.3390/clockssleep6040049

**Published:** 2024-11-18

**Authors:** Véronique-Aurélie Bricout, Sandro Covain, Jacob Paterno, Michel Guinot

**Affiliations:** 1 HP2, University Grenoble Alpes, F-38000 Grenoble, France; sandro.covain@univ-grenoble-alpes.fr; 2 INSERM U1300, HP2, F-38000 Grenoble, France; mguinot@chu-grenoble.fr; 3 CHU de Grenoble, HP2, UM Sports et Pathologies, F-38000 Grenoble, France; jpaterno@chu-grenoble.fr; 4 UM Sports et Pathologies, CHU Sud, CS 90338, F-38434 Echirolles, France

**Keywords:** sleep, sex/gender, children, physical activity, autism spectrum disorder

## Abstract

Physical activity (PA) programs have been found to result in improved sleep in males with autism spectrum disorder (ASD), but little is known about the female characteristics. The aim of this work was to assess sex differences in sleep and PA indices using an accelerometer over 7 days and 7 nights. Sleep and PA variables were measured with questionnaires and with accelerometry in twenty-four children with ASD (16 boys, 10.3 ± 2.8; 8 girls, 11.1 ± 3.9). Some significant differences were reported between girls and boys. The total time in bed and wake time after sleep onset (WASO) were significantly higher in girls compared to boys (*p* < 0.01), whereas sleep efficiency was significantly lower in girls (*p* < 0.01). The results obtained from the sleep questionnaire (CSHQ) show averages above the threshold of 41 in both groups (the threshold indicates the presence of sleep disorders or low sleep quality). The number of daily steps was significantly lower in the girls’ group (*p* < 0.01), and the PA volume for vigorous and strong vigorous intensities was significantly higher in the boys’ group (*p* < 0.01 and *p* < 0.05, respectively). Our results show major alterations in girls, with a low level of PA and sleep alteration. PA is a relevant non-pharmacological approach to improve sleep quality and achieve sufficient sleep duration. However, particularly for girls with ASD, more personalized approaches to improve sleep may be needed to manage specific associated disorders.

## 1. Introduction

Regular physical activity (PA) is recognized as a key factor in healthy development, allowing children who engage in regular physical activity to enjoy a positive health trajectory and a lifestyle that helps prevent or delay chronic diseases [1]. However, several studies have reported that most children do not reach the minimum recommendations for daily physical activity despite the benefits of regular PA in children [2,3]. For more than 10 years, it has been demonstrated that regular PA has a positive impact on the quality and quantity of children’s sleep in general, but the results are not all consensual, and the effects of PA on sleep vary. 

A meta-analysis published in 2015 [4] reported a positive impact on total sleep time, sleep onset latency, and slow wave sleep following acute and regular physical exercise but a negative impact on rapid eye movement sleep. More recently, the meta-analysis published by Atoui et al. [5] showed that these bidirectional “physical activity–sleep” effects were more complex as there was considerable variability from day to day but also according to the individual and the physical activity itself (frequency of PA, intensity, duration, and type of PA) [4,6,7,8]. Other studies have also reported that Wake After Sleep Onset (WASO) and sleep quality were significantly positively and negatively associated with physical activity at the intra-individual level. Sleep efficiency was positively associated with physical activity at the interindividual level.

There are significant interindividual differences in sleep and physical activity variables, which are even more dependent on age, gender, and any associated chronic illness or impairment [9,10,11]. In this context, children with autism spectrum disorder (ASD) are more likely to have impaired sleep than typically developing children. ASD is a complex neurodevelopmental disorder characterized by social communication impairments, repetitive behavior, and restricted interests [12]. 

Several studies suggest that sleep problems are one of the most common issues experienced in the ASD population, with an estimated prevalence of 50 to 80% [13,14,15]. The most frequently reported alterations are shorter total sleep duration induced by higher sleep onset latency, more frequent and longer nocturnal awakenings, early morning awakenings, and, consequently, lower sleep efficiency [16,17]. For children with ASD, improving the duration and quality of sleep is essential because sleep plays a key role in brain development and maturation. It is essential for the construction of the synaptic network, playing a significant role in flexibility and cognitive adaptation [18]. Moreover, sleep is vital for improving memory, behavioral regulation, cognition, and learning [19]. Other clinical signs are frequently reported in ASD, including deficits in basic abilities such as difficulties with expression, language, and interaction, as well as significant impairments in motor capacities, leading to reduced participation in physical activity [18,20,21]. 

To address sleep disorders in children with ASD, various pharmacological and/or non-pharmacological interventions have been evaluated. Emerging evidence suggests that PA can improve sleep in children with ASD by increasing total sleep duration and reducing awakenings [18,22,23]. PA reduces maladaptive patterns in children with ASD [24,25,26]. Nevertheless, it is still difficult to understand exactly how physical activity can influence sleep quality or quantity and vice versa [6]. Some associations have been reported between sleep loss and exercise-induced somatic symptoms [27], suggesting physiopathological interactions between sleep and somatic symptoms such as pain, fatigue, negative mood, dyscognition, somatic symptoms, and vigilance. Interest in the benefits of PA for children with ASD is growing. A recent meta-analysis in the ASD population reported that PA programs enhance sleep quality [22] and have additional benefits, including improving self-esteem, confidence, motivation, behaviors, and interactions, as well as promoting social connections and education [17,28].

In these studies, there are few results on the interindividual differences in such PA programs. In particular, not only is there very little research available on the differences in sleep patterns between girls and boys with ASD, but there is even less research on the benefits of physical activity programs on the frequency, type, or severity of sleep disorders in girls with ASD. ASD has long been considered more common in males than in females, with a sex ratio of 4:1 [29]. However, recent population-based epidemiological studies have shown that this ratio is more likely 2:1 [30]. Consequently, studying populations of girls too often ignored in previous studies is now a challenge. This is due to the under-representation of female participants and variations in sampling and methodology [31,32]. Overall, studies on young boys and girls with ASD display a similar pattern in autistic symptoms, and differences are limited in scope. In a recent review, authors reported that young boys with ASD presented more stereotyped and repetitive behaviors than young girls adjusted for cognitive and age status [22]. Nevertheless, other studies have reported no gender difference in depression [33], anxiety, obsessive–compulsive disorder, and bipolar disorder [34,35]. These results highlight the need to extend knowledge of a female phenotypic profile in autism to improve care and modify diagnostic tools adjusting for gender. 

Therefore, the aim of this work was to investigate the sleep indices (sleep durations and sleep quality indices) and physical activity levels (PA times according to intensity) by stratifying subjects based on gender. 

We hypothesize that girls have lower PA levels than boys regarding duration of vigorous PA and strong vigorous PA, which could explain the differences in sleep efficiency in this experimental group.

## 2. Results

The characteristics of the children are presented in Table 1. There was no statistical difference in the demographic characteristics between the boys’ and girls’ groups (Table 1). None of the children had visual or hearing impairments. One boy had skin hypersensitivity (refusal of skin-to-skin contact), and one girl had noise hypersensitivity, but for these two children, these observations were not a limitation for PA. All children contributed 7 nights and 7 days of accelerometry data for analysis, and the device was well tolerated in this study. Regarding sleep variables, some significant differences were observed between the two groups. The total time in bed and WASO were significantly higher in the girls’ group compared to boys’ group (*p* < 0.01), whereas sleep efficiency was significantly lower in the girls’ group (*p* < 0.01; Table 1). The results obtained from the sleep questionnaire (CSHQ) showed averages above the threshold of 41 in both groups, with 13 boys out of 16 (81.25%) and 6 girls out of 8 (75%). The PCA was applied to the total group on the five significant variables which helped explain 87.8% of the variance on the first two dimensions (50% and 37.8%), which was a very satisfactory outcome. 

Regarding PA variables, the main differences between the two groups were the PA volume for vigorous and strong vigorous intensities (*p* < 0.01 and *p* < 0.05, respectively) and the daily number of steps, which was significantly lower in the girls’ group (*p* < 0.01; Table 1).

**Figure 1:**
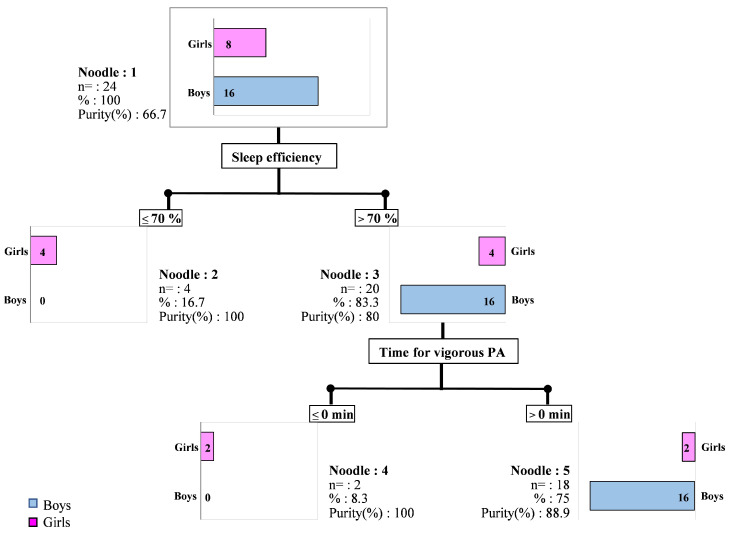
Classification tree for 24 children, with 10 variables analyzed as model input (1. gender; 2. total time in bed (h); 3. sleep efficiency (%); 4. WASO (min); 5. sedentary behavior (min); 6. Time for MVPA (min); 7 Time for moderate PA (min); 8. Time for vigorous PA (min); 9. Time for of strong vigorous PA (min); 10. daily step number) for three variables identified in classification: 1. gender, 2. sleep efficiency, and 3. duration of vigorous PA.

The classification tree revealed 3 classification variables out of the 10 analyzed in the model (Figure 1). This classification tree therefore made it possible to divide the children into two groups according to sleep efficiency: a group related to sex, and in a second noddle, a group related to the time of vigorous PA. The threshold value for the first node classification was sleep efficiency of 70%, whereas the reference value is 85%. In this classification, 4 girls with very low sleep efficiency values were grouped together, excluding boys. All the boys were in the >70% group. The second level of classification was dependent on the time of vigorous PA, with a distribution in which all the boys were still grouped together on the same cluster. 

**Figure 2:**
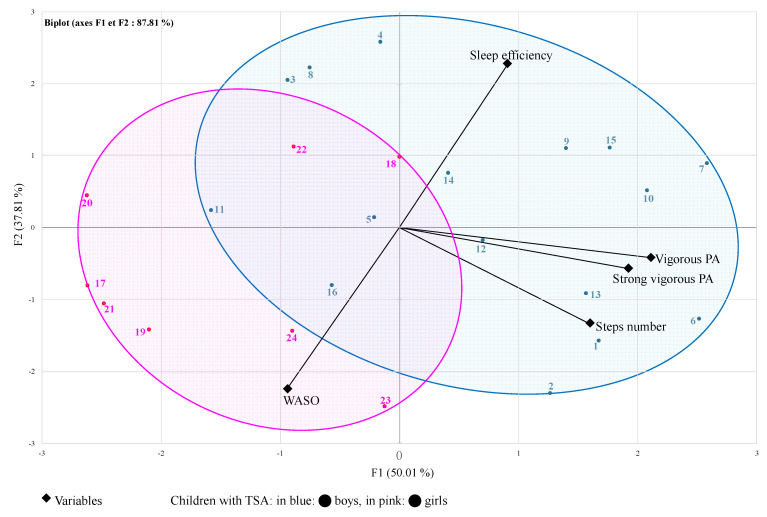
Principal component analysis (PCA) biplot. Children are represented from 1 to 24 (from 1 to 16 for boys and from 17 to 24 for girls) and 5 predictive variables (2 related to sleep and 3 related to PA).

In the biplot representation (Figure 2), a child on the same side of a given variable obtained the best score for this variable. A low value for this variable was attributed to a child on the opposite side. For example, a child positioned near an item related to PA variables (such as child #16) presented a very high number of daily steps (16,890 steps/day) and very high vigorous and strong vigorous PA durations (22 and 2 min/d, respectively). A child on the opposite side (such as child #22) presented a low number of steps (4573 steps/day) and low vigorous and strong vigorous PA durations (3 and 0 min/d, respectively). The upper right-hand quarter exclusively comprised boys with high sleep efficiency and few nocturnal awakenings, while the lower left-hand quarter included 5 girls and one boy with low sleep efficiency and very high nocturnal awakenings (Figure 2).

## 3. Materials and Methods

### 3.1. Sample

Twenty-four children with ASD were recruited from a local association and divided into two groups: boys (n = 16) and girls (n = 8; Table 1). For this study, we focused on sex as the biological difference between male and female as defined by the World Health Organization (Gender and Health (who.int)). Each subject and their parents received oral and written information, agreed to participate, and signed a consent form. This study was approved by the local ethics committee of the hospital (N°A00-865 40), with registration on the Clinicaltrials.gov registry (NCT: 02830022). Research was conducted according to the principles expressed in the Declaration of Helsinki. 

The ASD diagnosis was assessed by experienced physicians and psychologists according to the *Diagnostic and Statistical Manual of Mental Disorders*-fifth edition criteria [12] and also with the Autism Diagnostic Observation Schedule (ADOS) [36]. Intellectual Quotient (IQ) was determined using the Wechsler Intelligence Scale for Children, 4th edition [37]. The inclusion IQ criterion was children with an IQ > 70. Children with one or more complex neurological disorders (e.g., epilepsy), comorbid psychiatric disorders, currently taking medication likely to affect sleep (e.g., melatonin supplements), or with a contraindication to physical exercise were not included in the study. The children attended school regularly and were physically active for at least 30 min a day (leisure, playtime games, or performing supervised sports activities). 

### 3.2. Procedure 

A medical examination was carried out for each participant, and a resting electrocardiogram was prescribed to assess the absence of contra-indications to physical activity. Height and weight were measured, and body mass index was calculated (BMI: body weight in kg/height in m^2^).

Each participant wore an accelerometer (SenseWear^®^ Pro Armband 3, Bodymedia, Pittsburgh, PA, USA) during a 1-week assessment (7 consecutive days and 7 nights). Participants and parents completed a daily diary to distinguish periods when the participant did not wear the accelerometer (bathing or water activity). Such unworn time was then excluded from the analysis. The accelerometer used was a bi-axial system, worn on the right tricep triceps. It incorporates a variety of measured parameters (accelerometry, heat flux, galvanic skin response, skin temperature, and near-body temperature) and demographic characteristics (age, height, weight, and gender) into proprietary algorithms to estimate energy expenditure. Accelerometers have previously been found to reliably measure the PA of subjects with ID [38].

This device measures the total time in bed (h), total sleep duration (h), bedtime resistance (min), sleep latency (min), wake-up time resistance (min), awakening latency (min), wake after sleep onset (WASO, min) and sleep efficiency (calculated by the accelerometer as total sleep duration/time in bedx100, %). It was also used to calculate the time spent in sedentary, moderate, vigorous, or strong vigorous PA (min). 

Each child completed two questionnaires, if necessary, with the help of a parent, but none of the children mentioned any difficulty in completing them. The physical activity questionnaire for children (PAQ-C; [39]), one of the most widely used questionnaires, has acceptable reliability and convergent validity [40]. It includes 9 items graded from 1 to 5, providing a global estimation of the PA level and sedentary activities of the children over the past seven days. The score was calculated to estimate PA levels as follows: light (score = 1), moderate (score = 2–4), and vigorous (score = 5). The higher the value, the more active the subject.

One sleep questionnaire was also completed. The children’s sleep habits questionnaire (CSHQ), developed by Owens et al. [41] and completed by parents, is the most commonly used questionnaire to assess the sleep habits of children with ASD. It includes 45 items, and parents are asked to assess on a three-point scale whether the described behavior occurs “rarely” (0–1 times), “sometimes” (2–4 times), or “usually” (5–7 times) during a typical week. For each sleep-related difficulty subscale, the child receives a score indicating the extent of difficulties. All subscale scores can be combined to a total difficulty score. This score ranges from 33 to 99, with higher scores indicating more sleep-related difficulties. A total score of 41 (sensitivity of 0.80 and specificity of 0.72) was identified by the receiver operating characteristic (ROC) analysis as the clinical cut-off for sleep problems. If the total CSHQ score was higher than 41, it indicated the presence of sleep disorders or low sleep quality. 

## 4. Data Analysis

The data are expressed as the means ± standard deviation of 7 days or 7 nights, collected from the monitoring device and extracted with the Sensewear Software (Version 7.0, Pittsburgh, PA, USA). The average values for weekdays (Monday to Friday), for weekend days (Saturday and Sunday), and for the total days of the week were calculated. Firstly, to assess the differences between PA levels and sleep characteristics related to sex, several analyses were carried out. For each group, a repeated-measures analysis was performed, which did not reveal any differences from day to day, as the sleep data varied minimally from night to night for each child. A second analysis compared the weekday versus weekend averages but reported no difference. 

To compare the boys’ and girls’ groups, an independent samples T-test (for parametric data) and the Mann–Whitney test (for non-parametric data) were performed using an average recorded over the 7 days and nights (Software XLSTATS 2024, Lumivero, Denver, CO, USA). Significance was accepted when *p* < 0.05.

To investigate sleep indices and physical activity levels by stratifying subjects based on gender, two classification methods were used (a classification tree and a principal component analysis). Classification trees are methods used to deliver models that explain and predict the classification of individuals to a modality of a qualitative variable based on explanatory quantitative and qualitative variables. A principal component analysis (PCA) was performed on the data obtained using the accelerometer (R software^®^, version 4.0). This is one of the most frequently used methods for analyzing multivariate data. It is used to study multidimensional data sets with quantitative variables. The PCA was supplied with the normalized version of the original predictors. In this part of our study, we had n = 24 observations (24 children with ASD) and *p* = 15 predictors (7 predictors related to PA data and 8 predictors related to sleep data from actimetry). To comply with the PCA application rule, which is 5 observations for 1 predictor, we applied the elastic net method, which simultaneously permits the automatic selection of pertinent variables. It can also select groups of correlated variables [42]. The 5 predictive variables selected were (1) sleep quality index; (2) WASO; (3) duration of vigorous PA; (4) duration of strong vigorous PA; and (5) number of steps. 

## 5. Discussion

The aim of this work was to investigate sleep indices and physical activity levels by stratifying subjects based on gender.

Although PA plays a significant role in the development of children, very little is known about PA behavior and its possible link with sleep in children with ASD, particularly in girls with ASD. Some studies indicate that daytime PA is associated with the quantity and quality of sleep among healthy children and adolescents [43,44,45]. Nevertheless, in children with ASD, this relationship is not clear as the interactions between sleep and autism are complex and multi-causal. 

### 5.1. PA in ASD and Gender-Related Differences

The focus and the originality of our study are based on the combination of two simultaneous PA measurement methods: an accelerometer, which provides information about PA intensity and duration, and a PA questionnaire (PAQ-C). The accelerometer collects data on daytime and nighttime activity patterns and intensity as they occur in children’s daily lives over seven consecutive days and nights. All the children in our study tolerated the accelerometer well without any withdrawal or sensory problems, and the wearing times were very satisfactory in both groups, confirming perfect adherence to the study. An accelerometer is widely used to assess PA and sleep variables in children with ASD, and several studies have demonstrated its usefulness as it provides objective measures [38] as opposed to a subjective assessment using a questionnaire.

In our study, we observed that the evaluation of physical activity (PA) using the PAQ-C did not reveal any differences between the groups of girls and boys. This questionnaire did not appear to be sufficiently discriminative to assess gender differences in a population of children with ASD, likely because the PAQ-C lacks the precision required to assess varying PA intensities. In contrast, the accelerometry assessments showed significant gender differences. While the girls were twice as active as boys (although the difference was not statistically significant), they engaged in significantly less vigorous (*p* < 0.01) and very vigorous PA (*p* < 0.05). Additionally, they took significantly fewer steps per day (*p* < 0.01), falling below the recommended 10,000 steps per day. Numerous studies have reported lower levels of PA in cohorts of children with ASD compared to their typically developing peers of the same age, particularly in moderate-to-vigorous physical activity (MVPA), with increased sedentary behaviors [46,47,48]. 

A previous review reported that the time spent in MVPA by children with ASD ranged from 34 to 166 min per day [46]. In our study, all children fell within this range, but the girls averaged 129 ± 86 min per 24 h, while the boys were more active (152 ± 59 min per 24 h, NS), with significantly higher PA levels in more intense activities (×3.5 for vigorous and ×6.5 for strong vigorous PA). Although girls with ASD engaged in less PA than boys with ASD, they still adhered to the 2020 World Health Organization (WHO) Guidelines on PA and Sedentary Behaviors, which recommend that children aged 5 to 17 engage in at least 30 min of moderate-intensity aerobic activity five days a week or 20 min of vigorous-intensity aerobic activity three days a week [49,50].

Several studies have identified potential reasons for the lower PA levels in children with ASD, including a lack of sports clubs or associations, as well as a shortage of trained professionals to manage children. Additionally, there are many parental barriers, such as economic, time-related, and emotional challenges [51,52]. 

However, to date, no study has specifically examined the possible reasons unique to girls that could explain these limitations to physical activity. For example, is the choice of physical activities diverse enough to meet the interests and motivations of young girls? In line with our hypothesis that there are differences in PA levels between girls and boys with ASD, which could potentially explain the differences in sleep characteristics in this experimental group, it is essential to understand how PA levels can positively impact sleep.

### 5.2. Sleep in ASD and Gender-Related Differences

Measuring sleep in children with ASD requires objective methods beyond subjective questionnaires. Tools that are easy to use, home-based, non-invasive, and well tolerated by children are necessary. While polysomnography is the gold standard for assessing sleep patterns, it may be difficult for children with ASD to tolerate a hospital environment. Accelerometry, widely used in sleep research and clinical practice, provides an objective and reliable method based on detecting movement and rest [38]. In our study, all children wore the accelerometer consistently (>23 h/day in both groups), confirming that the device was well tolerated. All children with ASD had sleep durations below the pediatric recommendations (10 to 12 h of effective sleep for this age group [53], as well as low sleep efficiency, falling below the 85% threshold [54]), in line with the existing literature. Notably, these results showed more pronounced impairment in the girls’ group, as illustrated by the classification tree and PCA. These findings are consistent with previous studies [31,55,56]. In children with ASD, significant sleep disturbances are explained by shifts in the sleep–wake cycle, particularly increased sleep onset latency. Nighttime awakenings are more frequent, with difficulties initiating and maintaining sleep, fear of the dark, and long and frequent nocturnal awakenings [55,56]. Shorter total sleep times have also been observed in children with ASD, who tend to wake up earlier in the morning [55,56]. Frequent nighttime awakenings of varying durations lead to the activation of the sympathetic nervous system during deep slow-wave sleep, reducing the quality of recovery [55,56]. Consequently, children who wake up frequently and have less efficient sleep are more fatigued during the day, which contributes to increased behavioral problems. 

In fact, a shorter total sleep time is associated with increased stereotyped behavior and a greater decline in social skills [57]. Insufficient sleep is believed to contribute to daytime sleepiness, exacerbating behavioral problems in children with ASD, such as reduced attention, learning difficulties, memory problems, and inappropriate behavior [57]. In our study, we found that girls had significantly higher wake after sleep onset (WASO) (*p* < 0.01) and lower sleep efficiency compared to boys (*p* < 0.01). The few studies conducted on girls with ASD have yielded conflicting results. Some suggest that boys and girls exhibit similar developmental patterns with no differences in sleep characteristics [33,35], while others indicate that sleep disorders are more severe in girls and are associated with a greater number of co-occurring conditions (e.g., epilepsy, low IQ) [58]. A recent study by Estes et al. [59] found that girls with ASD (aged 6–12 years; evaluated by the CSHQ) showed increased bedtime resistance, sleep anxiety, sleepiness, and reduced sleep duration compared to boys with ASD, but there were no differences in sleep onset delay, WASO, or parasomnias. Our results are similar, but with accelerometry assessments, we found a significantly higher WASO in girls. It is possible that actimetry, as an objective sleep measure, is more accurate than questionnaires and provides clearer evidence of these sleep impairments in the girls’ group.

It also appears that poor sleep is associated with mental health issues in girls with ASD, particularly depression and anxiety, which can further negatively impact sleep characteristics [60]. Girls with ASD are significantly more likely to have anxiety disorders and sleep disturbances than boys with ASD [16]. Gotham et al. (2015) and Uljarević et al. (2019) reported that although boys with ASD are more prone to depression, girls with ASD tend to exhibit more depressive and anxious symptoms over time [61,62]. In our study, we did not directly assess anxiety or depression profiles, but the CSHQ questionnaire, which evaluates sleep difficulties such as sleep anxiety, bedtime resistance, and night wakings, indicated scores above 41, confirming the presence of sleep disorders in this group of children with ASD, with higher total scores in girls compared to boys. The CSHQ results also point to poor sleep quality, with scores exceeding the threshold of 41. Thus, we can describe our sample as “poor sleepers”.

### 5.3. Sleep and PA in ASD and Gender-Related Differences

Several studies have demonstrated a strong association between sleep quality and PA levels. Research has shown that for every additional hour of moderate-to-vigorous physical activity (MVPA) and increased intensity, there are beneficial effects on sleep [7]. Similar studies have also found that more MVPA and less sedentary behavior are linked to better sleep quality [47,48,63]. While this bi-directional relationship between sleep and physical activity has been established, no study has yet examined the gender effect on PA levels and sleep quality in a population of girls with ASD. All previously published results have been based on cohorts of boys, excluding girls from these studies.

In our study, we found alterations in sleep patterns in both groups, which were associated with significantly lower levels of vigorous and strong vigorous PA in the girls’ group compared to the boys’ group. The PCA classification made in this study further supports this, as the boys clustered together around the “sleep” and “physical activity” components (Figure 2; blue cluster). Moreover, all boys were grouped in the same classification tree (Figure 1) without PA intensity differentiating them. For the girls, we can assume that insufficient PA was the key discriminating factor.

Children with ASD are less active than their peers [23], and a sedentary lifestyle could also contribute to their sleep problems [64]. Poor sleep quality negatively affects the development and quality of life of children with ASD and disrupts family life. These results are even more pronounced in the girls’ group. While the girls spent more total time in bed, they had significantly higher WASO compared to boys, resulting in significantly lower sleep efficiency in the girls’ group (*p* < 0.001). Our results are therefore in line with the few studies available on gender-dependent sleep characteristics in children with ASD.

However, very few studies have explored the potential links between sleep and physical activity in girls with ASD. Smidt et al. reported some results using questionnaires to assess the relationships between sex, sleep, and PA [24,32]. They found a sex difference, with an association between PA and sufficient sleep duration apparent in males with ASD but not in females with ASD.

In ASD, possible effects of PA on sleep have been reported by several authors. PA volume might have a positive or negative effect on sleep quality [48,65,66]. It is essential to understand how PA levels could positively impact sleep. We hypothesize that insufficient PA may negatively affect both the quality and quantity of sleep in the girls’ group. A sedentary lifestyle is often proposed as a contributing factor to sleep alterations [23], a finding supported by other studies [7,40] which showed that more MVPA and less sedentary behavior are associated with better sleep quality.

Wachob and Lorenzi [21] have demonstrated that in children with ASD, increased PA is associated with improved sleep quality, and regular exercise represents a promising non-pharmacological treatment for sleep issues. Regular PA is particularly important to ensure quality sleep, especially in the context of autism, where sleep disorders are common. PA is a simple, cost-effective intervention that also offers significant benefits for core ASD symptoms. However, more research is needed to determine the optimal type, duration, and intensity of PA that should be recommended. In ASD, impairments in social interaction and communication may limit participation in certain activities, such as team sports. Furthermore, in children with ASD and with an IQ > 70 it is often reported that ASD is associated with marked clumsiness and motor skill disabilities [67,68], which complicates the ability to engage in physical activities such as running or walking [23,69,70]. These factors should be carefully considered, especially for girls who wish to participate in specific physical activities. 

Finally, our study shows some strengths and limitations. 

The strengths of this study include the use of objective methods to evaluate both sleep and PA, alongside questionnaires, which is a new finding for this population, and the inclusion of a sample that respects the 2:1 male-to-female ratio. The use of these two methods (objective and subjective) is important as it provides complementary information that cannot be assessed by one method alone.

However, to increase the statistical power and reliability of the classification-related results, there is a need for a larger sample size, particularly with more female participants. Additionally, since our study only included children with an IQ > 70, it is necessary to replicate this work with children who have more severe forms of ASD. In any case, more rigorous studies with strong methodologies are required to better understand the beneficial effects of exercise on sleep patterns, especially in girls compared to boys.

## 6. Conclusions

In our sample, we found major alterations in girls, for whom the low level of PA could, in part, be one of the elements of the sleep alteration associated with other characteristics linked to the autistic profile. Even if a large consensus exists regarding the fact that the daytime impact of PA is a major issue in sleep disorders, the nature and the magnitude of this impact are still controversial, specifically in girls with ASD. PA is a relevant non-pharmacological approach to improve sleep quality and sufficient sleep duration. However, for some groups of children with autism, particularly girls, more personalized approaches to improving sleep may be needed to manage specific associated disorders. Our study is a first step towards investigating the female phenotypes of ASD and underlines the importance of further research in this area.

## Figures and Tables

**Table 1:** Participants’ anthropometric characteristics; sleep and PA assessments related to gender.

		Girls (n = 8)	Boys (n = 16)
Demographic characteristics and questionnaires	Age	11.1 ± 3.9	10.3 ± 2.8
	Height (cm)	140.6 ± 14.9	143.2 ± 16.0
	Weight (kg)	38.1 ± 14.7	35.2 ± 9.9
	BMI (kg/m^2^)	18.5 ± 3.8	16.9 ± 2.3
	PAQ-C Score	2.1 ± 0.8	2.6 ± 0.7
	CSHQ Score	49.9 ± 9.7	47.2 ± 7.2
	Total time wearing of accelerometer (h)	23.4 ± 0.5	23.4 ± 0.3
Sleep characteristics	Total time in bed (h)	10.03 ± 0.96	9.18 ± 0.40 **
	Total sleep duration (h)	7.23 ± 0.73	7.24 ± 0.52
	Sleep efficiency (%)	72.4 ± 6.5	79.0 ± 3.0 **
	Bedtime resistance (min)	11.6 ± 12.7	4.3 ± 3.3
	Sleep latency (min)	12.2 ± 12.3	14.6 ± 8.3
	Wake-up time resistance (min)	1.2 ± 2.9	0.8 ± 1.6
	Awakening latency (min)	15.0 ± 5.3	15.3 ± 6.1
	WASO (min)	128.0 ± 43.4	80.9 ± 20.5 **
Physical activity characteristics	Total time for PA (min)	129.4 ± 86.5	338.3 ± 580.2
	Sedentary behavior (min)	1276.9 ± 92.4	1246.5 ± 77.4
	Time for MVPA (min)	129.0 ± 86.2	151.8 ± 58.9
	Time for moderate PA (min)	124.0 ± 83.4	134.2 ± 48.2
	Time for vigorous PA (min)	4.9 ± 6.1	17.4 ± 15.0 **
	Time for strong vigorous PA (min)	0.4 ± 0.5	2.6 ± 3.3 *
	Daily steps number	9363 ± 3097	12,369 ± 3405 **

Values are shown as means ± SD. Sleep efficiency = ratio of total sleep duration/total time in bed. BMI: body mass index. GPAQ: global physical activity questionnaire (the higher the value, the more active the subject). CSHQ: children’s sleep habits questionnaire. WASO: wake time after sleep onset. PA: physical activity. MVPA: moderate to vigorous physical activity. Significantly different from girls * *p* < 0.05 and, ** *p* < 0.01.

## Data Availability

The data presented in this study are available upon request from the corresponding author. The data are not publicly available due to the medical characteristics of these data that represent private data. There are no prior publications or submissions with any overlapping information, including studies and patients.
